# Yellow-Fever

**Published:** 1871

**Authors:** W. P. Reese

**Affiliations:** Selma, Ala.


### *Yellow-Fever.*

The following letter from Dr. W. P. Reese, of Selma, Ala., giving some original views in regard to the character of Yellow-Fever, will not be without interest to the profession. We hope soon to present to the readers of this Journal, the Doctor's views more in detail:

OFFICE, July 18th, 1871.

*Prof. W. F. Westmoreland, Atlanta, Ga.:*

DEAR DOCTOR:—A copy of the "Atlanta Medical and Surgical Journal," vol. IX., No. 1, has been received. In glancing over the articles in this spirited little Journal, my attention was arrested by an article which I supposed was from your pen : "Cerebro-Spinal Meningitis." Our Mobile brethren, as you are, perhaps, aware, call the disease, "Cerebro-Spinal Fever." What particularly arrested my attention, and called forth this communication, was, that "Cerebro-Spinal Meningitis," was (is) "a part and parcel of the same epidemic influence known as influenza."—epidemic influenza. The whole article is replete with *truth*. The undersigned endorses what you say, and will here re-produce a sentence which he wrote in 1853, after the close of the fatal "epidemic yellow-fever" which visited this city in the fall of that year. "Epidemic Yellow-fever," so-called, is one of the *forms* of epidemic influenza, same in kind, differing only in degree."

The same disease had prevailed, epidemically, for six weeks before there had been but one or two deaths preceded by black vomit. The writer constantly maintained this view throughout the epidemic, treated all of his cases upon the same principles, ignored the terrific *name* "Yellow-fever." *Names* have been—are now, the curse of our profession.

The notes of seventy-three (73) cases treated during this *awful* epidemic, are in reach of the writer; seven (7) only of these died. And here is the treatment in a nut-shell:

1. An emetic of ipecac.
2. Put the patients between *blankets and keep them warm.*
3. Warm teas for drinks.
4. Salines as indicated.

*Remarks.*—It won in that epidemic.

Very respectfully yours, in haste,

W. P. Reese, M.D., Selma, Ala.

